# (±)-(*rel*-3*R*,3′*R*)-1,1′-Dimethyl-3,3′-bipyrrolidine-2,2′-dithione

**DOI:** 10.1107/S1600536812043498

**Published:** 2012-10-27

**Authors:** Lee G. Madeley, Andreas Lemmerer, Joseph P. Michael

**Affiliations:** a Molecular Sciences Institute, School of Chemistry, University of the Witwatersrand, Private Bag 3, PO Wits, 2050 Johannesburg, South Africa

## Abstract

The asymmetric unit of the racemic title compound, C_10_H_16_N_2_S_2_, a *C*_2_-symmetric bis­(thiol­actam), contains one half-mol­ecule, the complete mol­ecule being generated by a twofold axis symmetry operation. The five-membered ring is nearly planar, with a maximum deviation of 0.025 (1) Å. In the crystal, the mol­ecules are linked *via* weak C—H⋯S inter­actions, forming infinite chains along the *b*-axis direction.

## Related literature

For related synthesis, see: Tamaru *et al.* (1978 ▶); Schroth *et al.* (2000 ▶).
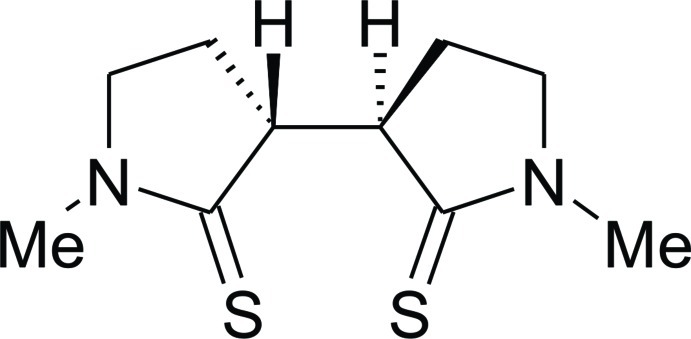


## Experimental

### 

#### Crystal data

C_10_H_16_N_2_S_2_
*M* *_r_* = 228.37
Monoclinic, 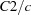

*a* = 20.520 (3) Å
*b* = 5.7237 (7) Å
*c* = 11.220 (2) Å
β = 122.009 (5)°
*V* = 1117.4 (3) Å^3^
*Z* = 4
Mo *K*α radiation
μ = 0.44 mm^−1^
*T* = 173 K
0.45 × 0.42 × 0.16 mm

#### Data collection

Bruker APEXII CCD area-detector diffractometer
Absorption correction: multi-scan (*SADABS*; Sheldrick, 1996 ▶) *T*_min_ = 0.827, *T*_max_ = 0.933
1759 measured reflections
1022 independent reflections
957 reflections with *I* > 2σ(*I*)
*R*_int_ = 0.016

#### Refinement

*R*[*F* ^2^ > 2σ(*F* ^2^)] = 0.030
*wR*(*F* ^2^) = 0.077
*S* = 1.09
1022 reflections
65 parameters
H-atom parameters constrained
Δρ_max_ = 0.22 e Å^−3^
Δρ_min_ = −0.21 e Å^−3^

### 

Data collection: *APEX2* (Bruker, 2005 ▶); cell refinement: *SAINT-Plus* (Bruker, 2004 ▶); data reduction: *SAINT-Plus* and *XPREP* (Bruker 2004 ▶); program(s) used to solve structure: *SHELXS97* (Sheldrick, 2008 ▶); program(s) used to refine structure: *SHELXL97* (Sheldrick, 2008 ▶); molecular graphics: *ORTEP-3 for Windows* (Farrugia, 1997 ▶) and *DIAMOND* (Brandenburg, 1999 ▶); software used to prepare material for publication: *WinGX* (Farrugia, 1999 ▶) and *PLATON* (Spek, 2009 ▶).

## Supplementary Material

Crystal structure: contains datablock(s) global, I. DOI: 10.1107/S1600536812043498/bt6851sup1.cif

Structure factors: contains datablock(s) I. DOI: 10.1107/S1600536812043498/bt6851Isup2.hkl

Supplementary material file. DOI: 10.1107/S1600536812043498/bt6851Isup3.mol

Supplementary material file. DOI: 10.1107/S1600536812043498/bt6851Isup4.cml

Additional supplementary materials:  crystallographic information; 3D view; checkCIF report

## Figures and Tables

**Table 1:** Hydrogen-bond geometry (Å, °)

*D*—H⋯*A*	*D*—H	H⋯*A*	*D*⋯*A*	*D*—H⋯*A*
C5—H5*B*⋯S1^i^	0.98	2.98	3.8373 (18)	146

Symmetry code: (i) 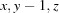.

**Fig. 1.**
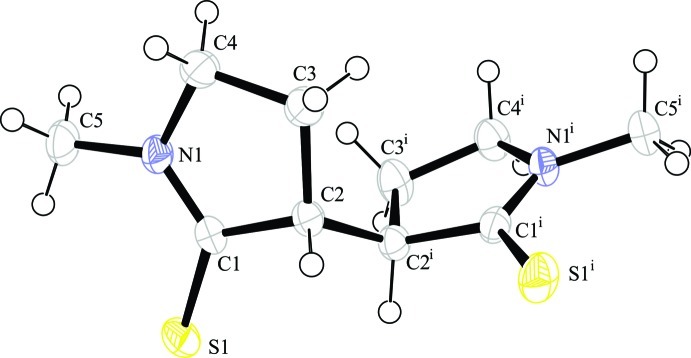
The asymmetric unit of (I) showing the atomic numbering scheme. Displacement ellipsoids are shown at the 50% probability level. Atoms with superscript (i) are at the symmetry position (-x, y, -z + 1/2).

**Fig. 2.**
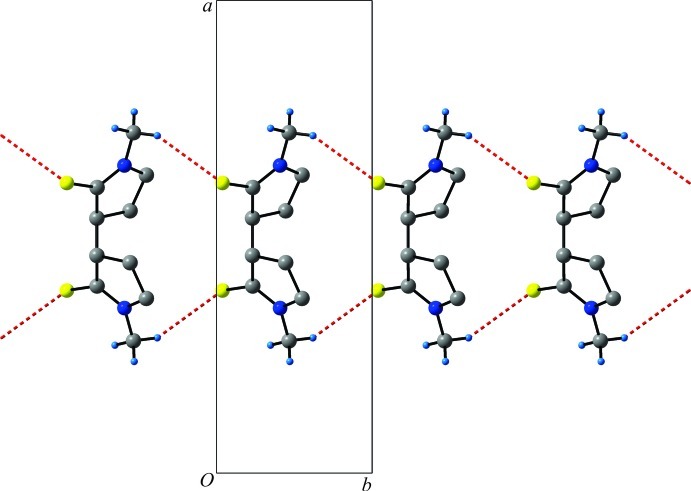
View of the hydrogen bonded chains of (I). C—H···S are shown as dashed red lines.

## supplementary crystallographic information

### Comment

The title compound, (±)-(*rel*-3*R*,3'*R*)-1,1'-dimethyl-3,3'- bipyrrolidine-2,2'-dithione, was obtained as a minor product from the attempted S_RN_1 arylation of deprotonated 1-methylpyrrolidine-2-thione with 4-bromoanisole under photolytic conditions. Previous workers had reported the same dimer from the reaction of deprotonated 1-methylpyrrolidine-2-thione with molecular iodine – apparently incorrectly as the *meso* diastereomer (Tamaru *et al.*, 1978), later corrected to the C_2_-symmetric isomer (Schroth *et al.*, 2000).

The asymmetric unit of the title compound consists of half a molecule around a twofold axis, and Fig. 1 shows the atomic numbering scheme. The complete molecule is generated by the twofold axis. Both stereogenic centres have the same relative configuration, which is depicted in Fig. 1 as *rel*-(*R,R'*). The opposite enantiomer in the crystal is generated by the c-glide in *C*2/c. The hydrogen bonding of the title compound consists of weak C—H···S hydrogen bonds from the methyl group to generate hydrogen bonded chains along the *b*-axis by unit cell translations only (Fig. 2).

### Experimental

A solution of 1-methylpyrrolidine-2-thione (580 mg, 5.5 mmol) in dry tetrahydrofuran (20 ml) was treated at 0 °C with a solution of *n*-butyllithium in hexane (5.5 mol). After 20 min 4-bromoanisole (690 µl, 5.5 mmol) was added, and the solution was irradiated for 30 min with a mercury lamp (125 W). The reaction mixture was poured into aq. NH_4_Cl solution, and the organic components were extracted with dichloromethane. Chromatography of the residue on silica gel after evaporation of the solvent returned unreacted 4-bromoanisole (50%), thiolactam (38%) and (±)-(*rel*-3*R*,3'*R*)-1,1'- dimethyl-3,3'-bipyrrolidine-2,2'-dithione (104 mg, 18%). The product was recrystallized from acetone to give colourless cubes, m.p. 476–477 K.

### Refinement

The C-bound H atoms were geometrically placed (C—H bond lengths of 0.99 for methylene CH_2_ and 0.98 for methyl CH_3_) and refined as riding with *U*_iso_(H) = 1.2*U*_eq_(C) or 1.5*U*_eq_(C).

### Crystal data

**Table:**

C_10_H_16_N_2_S_2_	*F*(000) = 488
*M**_r_* = 228.37	*D*_x_ = 1.357 Mg m^−^^3^
Monoclinic, *C*2/*c*	Mo *K*α radiation, λ = 0.71073 Å
Hall symbol: -C 2yc	Cell parameters from 419 reflections
*a* = 20.520 (3) Å	θ = 3.8–30°
*b* = 5.7237 (7) Å	µ = 0.44 mm^−^^1^
*c* = 11.220 (2) Å	*T* = 173 K
β = 122.009 (5)°	Cube, colourless
*V* = 1117.4 (3) Å^3^	0.45 × 0.42 × 0.16 mm
*Z* = 4	

### Data collection

**Table:**

Bruker APEXII CCD area-detector diffractometer	957 reflections with *I* > 2σ(*I*)
ω scans	*R*_int_ = 0.016
Absorption correction: multi-scan (*SADABS*; Sheldrick, 1996)	θ_max_ = 25.5°, θ_min_ = 2.3°
*T*_min_ = 0.827, *T*_max_ = 0.933	*h* = −19→23
1759 measured reflections	*k* = −6→6
1022 independent reflections	*l* = −13→5

### Refinement

**Table:**

Refinement on *F*^2^	0 restraints
Least-squares matrix: full	H-atom parameters constrained
*R*[*F*^2^ > 2σ(*F*^2^)] = 0.030	*w* = 1/[σ^2^(*F*_o_^2^) + (0.0336*P*)^2^ + 0.8972*P*] where *P* = (*F*_o_^2^ + 2*F*_c_^2^)/3
*wR*(*F*^2^) = 0.077	(Δ/σ)_max_ < 0.001
*S* = 1.09	Δρ_max_ = 0.22 e Å^−^^3^
1022 reflections	Δρ_min_ = −0.21 e Å^−^^3^
65 parameters	

### Special details

**Table:**

Geometry. All e.s.d.'s (except the e.s.d. in the dihedral angle between two l.s. planes) are estimated using the full covariance matrix. The cell e.s.d.'s are taken into account individually in the estimation of e.s.d.'s in distances, angles and torsion angles; correlations between e.s.d.'s in cell parameters are only used when they are defined by crystal symmetry. An approximate (isotropic) treatment of cell e.s.d.'s is used for estimating e.s.d.'s involving l.s. planes.

### Fractional atomic coordinates and isotropic or equivalent isotropic displacement parameters (Å^2^)

**Table:**

	*x*	*y*	*z*	*U*_iso_*/*U*_eq_	
C1	0.60416 (8)	0.7676 (3)	0.29080 (15)	0.0237 (3)	
C2	0.53949 (8)	0.7690 (3)	0.31920 (15)	0.0238 (3)	
H2	0.544	0.9137	0.373	0.029*	
C3	0.55488 (10)	0.5558 (3)	0.41423 (18)	0.0342 (4)	
H3A	0.5591	0.6041	0.5028	0.041*	
H3B	0.5127	0.4406	0.366	0.041*	
C4	0.63050 (9)	0.4508 (3)	0.44413 (17)	0.0318 (4)	
H4A	0.6706	0.4624	0.5453	0.038*	
H4B	0.6238	0.2846	0.4155	0.038*	
C5	0.72017 (9)	0.5339 (3)	0.36087 (19)	0.0343 (4)	
H5A	0.7294	0.6527	0.3086	0.051*	
H5B	0.7139	0.3809	0.3168	0.051*	
H5C	0.764	0.5289	0.4582	0.051*	
N1	0.65091 (7)	0.5918 (2)	0.35941 (13)	0.0254 (3)	
S1	0.61374 (2)	0.96194 (8)	0.18993 (4)	0.03401 (17)	

### Atomic displacement parameters (Å^2^)

**Table:**

	*U*^11^	*U*^22^	*U*^33^	*U*^12^	*U*^13^	*U*^23^
C1	0.0208 (7)	0.0274 (8)	0.0243 (7)	−0.0042 (6)	0.0130 (6)	−0.0032 (6)
C2	0.0227 (8)	0.0261 (8)	0.0265 (7)	0.0000 (6)	0.0157 (6)	0.0012 (6)
C3	0.0330 (9)	0.0395 (10)	0.0391 (9)	0.0072 (7)	0.0252 (8)	0.0136 (7)
C4	0.0318 (9)	0.0333 (9)	0.0366 (9)	0.0046 (7)	0.0225 (7)	0.0090 (7)
C5	0.0244 (9)	0.0406 (10)	0.0429 (10)	0.0030 (7)	0.0213 (7)	−0.0010 (7)
N1	0.0211 (6)	0.0296 (7)	0.0294 (6)	0.0014 (5)	0.0160 (5)	0.0015 (5)
S1	0.0327 (3)	0.0375 (3)	0.0394 (3)	−0.00300 (17)	0.0242 (2)	0.00804 (17)

### Geometric parameters (Å, º)

**Table:**

C1—N1	1.320 (2)	C3—H3B	0.99
C1—C2	1.520 (2)	C4—N1	1.4672 (19)
C1—S1	1.6711 (15)	C4—H4A	0.99
C2—C3	1.539 (2)	C4—H4B	0.99
C2—C2^i^	1.539 (3)	C5—N1	1.451 (2)
C2—H2	1	C5—H5A	0.98
C3—C4	1.526 (2)	C5—H5B	0.98
C3—H3A	0.99	C5—H5C	0.98
			
N1—C1—C2	109.09 (12)	N1—C4—C3	104.31 (12)
N1—C1—S1	126.33 (12)	N1—C4—H4A	110.9
C2—C1—S1	124.58 (11)	C3—C4—H4A	110.9
C1—C2—C3	105.06 (12)	N1—C4—H4B	110.9
C1—C2—C2^i^	110.96 (14)	C3—C4—H4B	110.9
C3—C2—C2^i^	114.74 (10)	H4A—C4—H4B	108.9
C1—C2—H2	108.6	N1—C5—H5A	109.5
C3—C2—H2	108.6	N1—C5—H5B	109.5
C2^i^—C2—H2	108.6	H5A—C5—H5B	109.5
C4—C3—C2	106.00 (13)	N1—C5—H5C	109.5
C4—C3—H3A	110.5	H5A—C5—H5C	109.5
C2—C3—H3A	110.5	H5B—C5—H5C	109.5
C4—C3—H3B	110.5	C1—N1—C5	125.84 (13)
C2—C3—H3B	110.5	C1—N1—C4	115.37 (12)
H3A—C3—H3B	108.7	C5—N1—C4	118.76 (13)
			
N1—C1—C2—C3	−1.74 (17)	C2—C1—N1—C5	−179.01 (14)
S1—C1—C2—C3	178.97 (11)	S1—C1—N1—C5	0.3 (2)
N1—C1—C2—C2^i^	−126.30 (10)	C2—C1—N1—C4	−1.04 (18)
S1—C1—C2—C2^i^	54.41 (12)	S1—C1—N1—C4	178.24 (11)
C1—C2—C3—C4	3.64 (17)	C3—C4—N1—C1	3.37 (18)
C2^i^—C2—C3—C4	125.78 (16)	C3—C4—N1—C5	−178.51 (14)
C2—C3—C4—N1	−4.13 (17)		

Symmetry code: (i) −*x*+1, *y*, −*z*+1/2.

### Hydrogen-bond geometry (Å, º)

**Table:**

*D*—H···*A*	*D*—H	H···*A*	*D*···*A*	*D*—H···*A*
C5—H5*B*···S1^ii^	0.98	2.98	3.8373 (18)	146

Symmetry code: (ii) *x*, *y*−1, *z*.
